# The Effect of Different Doses of Melatonin on in Vitro Maturation of Human Follicular Fluid-Derived Oocyte-Like Cells

**DOI:** 10.5935/1518-0557.20210086

**Published:** 2022

**Authors:** Saeed Azandeh, Mahin Taheri Moghadam, Mohammad Rashno, Mahvash Zargar, Pardis Abed Zadeh

**Affiliations:** 1 Cellular & Molecular Research Center, Medical Basic Sciences Research Institute, Department of Anatomical Science Faculty of Medicine, Ahvaz Jundishapur University of Medical Science, Ahvaz, Iran; 2 Department of Gynecology and Obstetrics, Fertility, Infertility and Perinatology Center, Imam Khomeini Hospital, Ahvaz Jundishapur University of Medical Sciences, Ahvaz, Iran

**Keywords:** Follicular fluid, melatonin, stem cell, oocyte like cell, infertility

## Abstract

**Objective:**

Human follicular fluid (FF) contains different cell populations including mesenchymal stem cells. Studies tried to improve their differentiation to oocyte and use them in infertility treatments. Using an antioxidant may improve the quality of these cells. The present study investigated the effects of different doses of melatonin on FF-derived cells grown to oocyte-like cells (OLC).

**Methods:**

Cell viability (MTT assay), flow cytometry, and ICC staining were utilized to evaluate CD105 and CD34 expression; colony forming unit assay (CFU-F) capability, qRT-PCR were used to investigate ZP1, ZP2, ZP3, GDF9, and SCP3 expression. AMH, Estradiol and Progesterone levels in the supernatant were measured. Morphological characteristics of fibroblast-like cells changing to a round shape were seen specifically in the group treated with melatonin 10^-7^M after 2 weeks.

**Results:**

There was no difference between control and treatment groups for MTT and CFU assays. ICC staining was positive for CD105 marker and negative for CD34 hematopoietic stem cell marker. qRT-PCR results indicated that ZP1, ZP2, GDF9, and SCP3 expression increased in the group treated with melatonin 10^-7^M in Week 2, while ZP3 decreased in this group. Progesterone and AMH were detected in differentiation medium.

**Conclusions:**

Melatonin may improve in vitro formation of OLCs.

## INTRODUCTION

With a prevalence of 9%-18% in the general population, infertility is a considerable problem since parenthood is one of the most desirable aims in the lives of many couples (Aghajanova *et al*., 2017). Societal changes provoked by a greater focus on education and careers have led to a change in the concept of childbearing. While the reproductive age of women has increased, reproductive capability has declined. Researchers have tried to find a way to overcome this limitation. The use of assisted reproductive technology (ART) has increased in recent decades (Talaulikar & Arulkumaran, 2012; Crawford & Steiner, 2015). During ICSI/IVF, oocytes are collected by transvaginal ultrasound-guided aspiration of Follicular Fluid (FF) (Aragona *et al*., 2011; Hussein *et al*., 2017). In vitro fertilization provides a new field of study by focusing on the analysis of FF. According to previous studies, FF contains different cell populations including steroidogenic cells, ovarian structural cells, and epithelial cells (Kollmann *et al*., 2017). Fertility clinics produce a large volume of waste follicular aspirate that might become an important source for potential research, diagnostics, and cell therapy in the future, since subpopulations of aspirated follicular cells might express some stem cell characteristics (Dzafic *et al*., 2014). Recent advances in cellular therapies have shed light on how stem cells can give rise to gametes and oocyte-like cells (OLCs) (Costa *et al*., 2018). Nowadays, researchers are trying to find ways to improve OLCs and obtain functional oocytes in vitro (Virant-Klun *et al*., 2013).

During IVF, free radicals produced by Reactive Oxygen Species (ROS) reduce oocyte quality. Using an antioxidant may decrease the resulting oxidative stress and improve outcomes (Vitale *et al*., 2016). Evidence shows a potential role for melatonin in controlling ROS-generating and detoxification/antioxidant genes as upstream events in cellular protection and anti-apoptotic mechanisms of mesenchymal stem cell (MSC), with possible positive effects as a free radical scavenger protecting oocytes (Tamura *et al*., 2008; Luchetti *et al*., 2014). Melatonin, the main product of the pineal gland, is an omnipresent molecule that modulates various mechanisms in the body. Because of its amphiphilic nature, it can pass through all morphophysiological barriers. It is also known as a greatly effective antioxidant (Cruz *et al*., 2014). Higher levels of melatonin have been observed in human follicular fluid than in serum, suggesting uptake of circulating melatonin by the ovaries. An affirmative correlation has been described between follicular fluid melatonin levels, in vitro fertilization outcomes, and oocyte quality (Tong *et al*., 2017; Olcese, 2020). Previous studies also showed melatonin's beneficial influence on implantation and oocyte maturation in vitro (Kim *et al*., 2013). In this study, we examined the effect of melatonin as a regulator of MSC differentiation to OLCs and as an antioxidant to reduce oxidative stress.

## MATERIAL AND METHODS

### Collection of human FF and cell culture

Human ovarian follicular fluid (FF) was collected from women (n=110) undergoing IVF treatment at the fertility center of AJUMS general hospital. The Ethics Committee of the Research Deputy of Ahvaz University of Medical Sciences (IRAJUMSABHC.REC1397.007) approved this study. During the procedure, an operator collected FF into sterile conical centrifuge tubes containing one drop of heparin. Subsequently, they were transported to the laboratory within 1 h. The hypo-osmotic technique described by Lobb & Younglai (2006) was used to remove red blood cells. FF samples were centrifuged at 1400 rpm for 6 min; 9.0 mL of sterile distilled water was added to the buffy coat containing follicular fluid cells and the tube was capped and mixed. After 60s, 1.0mL of 10x concentrated phosphate buffer saline (PBS; pH≈7) was added and mixed again. The tubes were centrifuged at 800 rpm for 3 min for ﬁnal collection. Isolated cells were cultured in 4-well plates with HG-DMEM (Dulbecco's Modified Eagle Medium) containing 15% FBS (fetal bovine serum) supplemented with amphotericin and penicillin plus streptomycin with different concentration of melatonin (Sigma-Aldrich, M5250) (10^-7^,10^-9^,10^-11^ M); the samples and kept in a 5% CO_2_ incubator at 37ºC for a maximum of 14 days, depending on the assay protocol.

### Cell viability

Right after the FF samples had been transferred to the laboratory, cell viability was analyzed with trypan blue staining (0.4%), so that the validity of the subsequent experiments could be ascertained. The cells were collected as described and 10µl of trypan blue was added to 10 µl of cells and DMEM suspension. This test is used to determine the proportion of viable cells in a cell suspension. It is based on the principle that living cells have selectively permeable cell membranes that exclude certain dyes, such as trypan blue, while dead cells do not. Therefore, the dead cells can be distinguished by their blue cytoplasm. Cells were observed with an inverted microscope (Leica; FA M205 Leica Microsystems) (Strober, 2015).

### MTT cell proliferation assay

Cell viability was determined after a week through tetrazolium salt MTT [3-(4, 5 dimethylthiazol-2-yl)-2, 5-diphenyltetrazolium bromide] assays. Cells were plated into 96-well culture plates. One week after the start of treatment with melatonin, 100 µl MTT (Sigma) was added to each well and incubated at 37°C for 4 h. The medium was gently aspirated, and then 100 µl DMSO were added to each well to dissolve formazan crystals. The optical density of each sample was then measured in a multi-well spectrophotometer at 570 nm (Phonchai *et al*., 2019).

### Flow cytometry

The presence of MSCs in FF was corroborated based on CD marker expression. To evaluate stem cell marker expression after 48h of culture, the cells were washed three times in PBS, detached with Trypsin-EDTA, centrifuged for 10 min at 1200 rpm, and rewashed in PBS supplemented with 1% Fetal Bovine Albumin. Positive controls were performed using HT29 (human colon adenocarcinoma cell line). The cells were incubated for 30 min with Anti-human CD105 PE conjugate (eBioscience,12-1057-42) in the dark at 4ºC. The samples were centrifuged for 10 min at 1200 rpm and PBS was added. The cells were analyzed on the same day with a BD FACSCalibur flow cytometer and the Cell Quest (Macintosh) software package (Dayer *et al*., 2017).

### Immunostaining

After 48h, cultured cells were washed in PBS three times and fixed in 4% paraformaldehyde for 10 min. After rewashing in PBS, the cells were permeabilized with tritonX100 at a concentration of 0.1% for 10 min. Then, nonspecific bindings were blocked with incubation in BSA 1% for 30min. Anti-human CD34 FITC conjugated antibody (eBioscience, 11-0349-42) and Anti-Human CD105 PE-conjugated antibody (eBioscience, 12-1057-42) diluted 2:100 and cells were incubated at 4ºC overnight. DAPI (4',6-diamidino-2-phenylindole) was used to counterstain and observe the nuclei (Alizadeh *et al*., 2019).

### Adipogenic and osteogenic differentiation

The multipotency of FF-aspirated cells was observed via their ability to differentiate into adipocytes and osteocytes. For adipogenic induction, cells were cultured in adipogenic medium containing 10% FBS, 10 µl Dexamethasone, 2.5 mg Indomethacin, 2.5 mg L-Ascorbic acid 2- phosphate in LG-DMEM for 14 days. The occurrence of intracytoplasmic lipid droplets was assessed in differentiated cells after Oil Red O staining. To promote osteogenic differentiation, cells were cultured in LG-DMEM supplemented with 10% FBS, 2.5 mg/ml Ascorbic acid 2-phosphate, 10 µl Dexamethasone and 500 µl β-Glycerol phosphate. Calcium aggregations were evaluated after Alizarin Red staining. The cells were observed in an inverted microscope (Leica; FA M205 Leica Microsystems) (Riva *et al*., 2014).

### Clonogenic Capacity

Since MSCs are known as colony-forming cells, the colony-forming unit (CFU) assay was performed to verify this claim. FF cells were seeded on a 4-well plate. After 2 weeks, the cultures were washed with PBS, fixed with 10% formalin, washed rapidly, and stained with 0.05% crystal violet. After 30 minutes at room temperature, the plates were washed and dried. Created colonies were observed in an inverted microscope using the DP2 BW soft imaging system. Colony size variation was assessed using software package image j (Eslaminejad *et al*., 2006).

### RNA extraction, cDNA synthesis, and real-time polymerase chain reaction

To analyze some of the OLC gene expression, total RNA was isolated on day 0, week 1, and week 2 using Rosch Total RNA Extraction kit according to manufacturer instructions. Purity and concentration were examined using a Nano Drope 2000c photometer. RNA reverse transcription into complementary DNA was performed using Yekta Tajhiz cDNA synthesis kits, which include Random Hexamer, dNTP, Rnase inhibitor, M-MLV; after the addition of 1 microgram of RNA, the samples were left for 1 h at 60◦C, ending in 5-minute cycles at 70◦C in an Eppendorf Master Cycler. qRT-PCR mixtures were set up in 10 µl of solutions containing 1 µl template, 5 µl SYBR green master mix, and distilled water for all samples. Reactions were optimized to implement maximum amplification efficiency for each gene, and amplicon size was checked by electrophoresis in 2% agarose gels. For ZP1, ZP2, and GDF9 samples, 0.2 µl of forward primer and 0.2 µl of reverse primer were used; for ZP3 and SCP3 samples, 0.4 µl of forward primer and 0.4 µl of reverse primer were used based on primer optimization data. The real-time PCR reaction was performed using the following program: 10 minutes for initial denaturation at 95ºC, then 15 s at 95ºC, 60ºC for 60 s, and 72ºC for 60 s. Glyceraldehyde-3-phosphate dehydrogenase (GAPDH) expression was used as the internal control gene. The sequences of primers are listed in Table1. The CT values represent the cycle number at which a fluorescent signal rose statistically above background.

**Table 1.** qRT-PCR primers.

GENES	PRIMER SEQUENCES		
**ZP_1_**	**Forward**	**CATTCAGGCATCCATTTTCC**	**(Canosa *et al*., 2017)**
	**Reverse**	**ACGAGCTGAAGGTCTCGTCT**	
**ZP_2_**	**Forward**	**GTGGAACGTTGTCGTGGATG**	**-**
	**Reverse**	**CCTGTGGCAGGCTAGAGAG**	
**ZP_3_**	**Forward**	**AGACCAGAATGCCTCCCCTT**	**-**
	**Reverse**	**TGCTTCTTCTGTCACATGTTTCT**	
**GDF_9_**	**Forward**	**TGTTCGGCTCTTCACCCC**	**(Yamamoto *et al*., 2002)**
	**Reverse**	**AGGATTCCTGTTACCTGGTCTCC**	
**SCP3**	**Forward**	**TCAAAGGCAGAAGCTTAACCAA**	**(Jørgensen *et al*., 2012)**
	**Reverse**	**CTTGCTGCTGAGTTTCCATCA**	
**GAPDH**	**Forward**	**ACTAACCCTGCGCTCCTG**	**(Danafar *et al*., 2018)**
	**Reverse**	**CCCAATACGACCAAATCAGA**	

### Hormone measurement

The cell culture mediums for the four groups were collected on week 1 and week 2 to assess OLC functional activity. Mediums Estradiol (E2) and Progesterone (P4) were determined using Estradiol Monibind and Progesterone Monibind respectively according to manufacturer instructions. For E2 and P4 measurement, 25 µl of sample with Biotin conjugation were incubated for 30 min at room temperature. Another of 50 µl conjugated enzyme were added and the samples were incubated for another 90 min and washed with washing buffer. After the addition of 100 µl of substrate to each well and 20 min of incubation in a dark room, 50 µl of stop solution was added. Sample absorption was measured at 450 nm with an ELISA reader (Mini Vidas). AMH level evaluation was performed using Vidas (Biomerieux) ready-to-use kits. The assay principle combines a one-step enzyme immunoassay sandwich method with final fluorescence detection (Sova *et al*., 2019).

### Statistical Analysis

Subpopulation and time variables were assessed via analysis of variance (ANOVA) and Tukey's multiple comparisons using GraphPad Prism software. A p-value <0.05 was considered statistically significant. SEM is shown in error bars.

## RESULTS

### Morphological analysis

**Figure 1:**
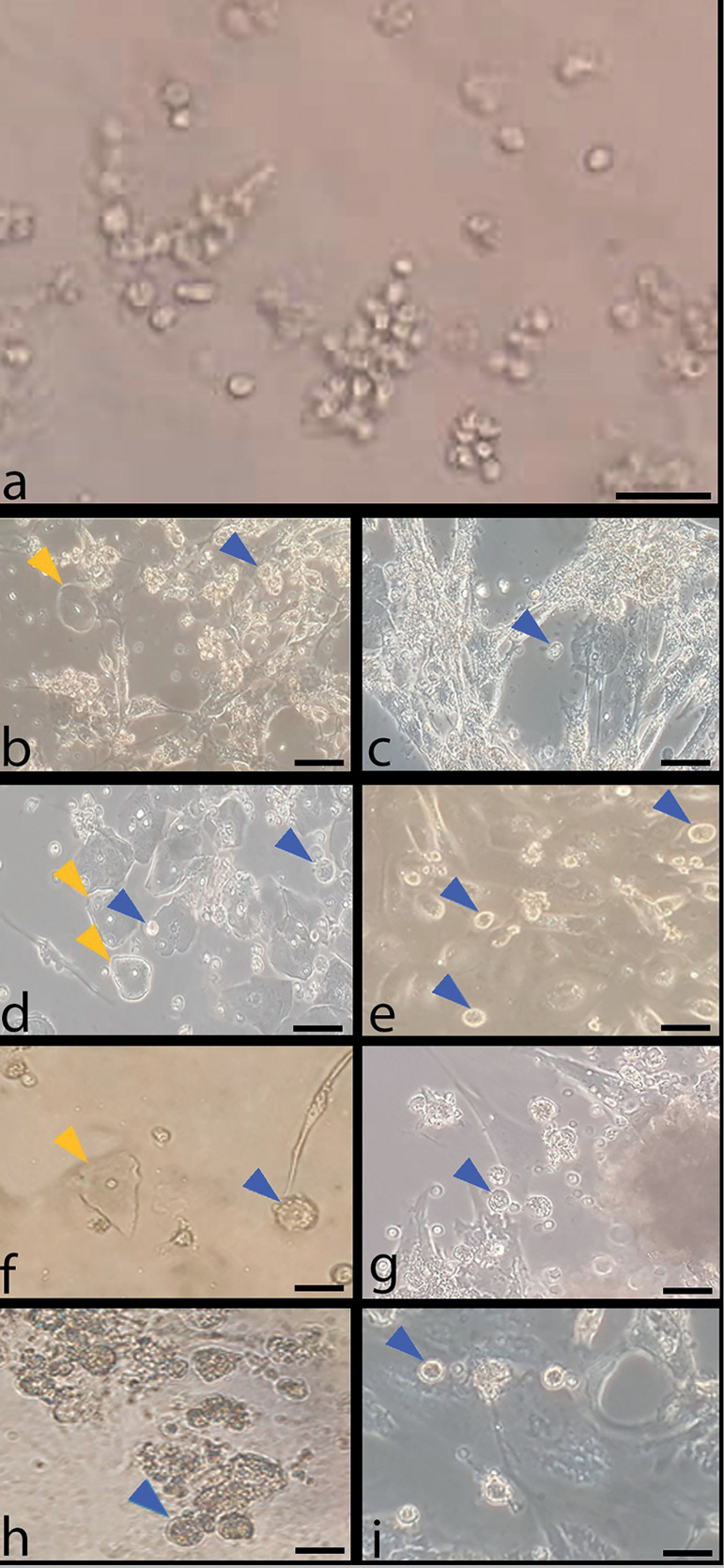
Morphological changes of follicular fluid cells in minimal culture condition. Control groups without melatonin treatment on day 0(a), week 1(b), week 2(c); groups treated with melatonin 10^-7^M on week 1(d) and week 2 (e); groups treated with melatonin 10^-9^M on week 1(f) and week 2(g); groups treated with melatonin 10^-11^M on week 1(h) and week 2(i); blue arrows: OLCs; yellow arrows: epithelial cells; 20 X.

A small rounded shape floating cell population was present on Day 0 as heterogeneous cells displaying elongated fibroblast-like shape, flattened epithelial-like shape, and neuron-like shape cells began to appear. OLCs also appeared a few days into in vitro culture and growth was observed for 2 weeks. Based on microscopy images, the best and most exuberant formation of OLCs occurred in the groups treated with 10^-7^ M melatonin (Figure 1).

### Cell viability and Proliferation

**Figure 2:**
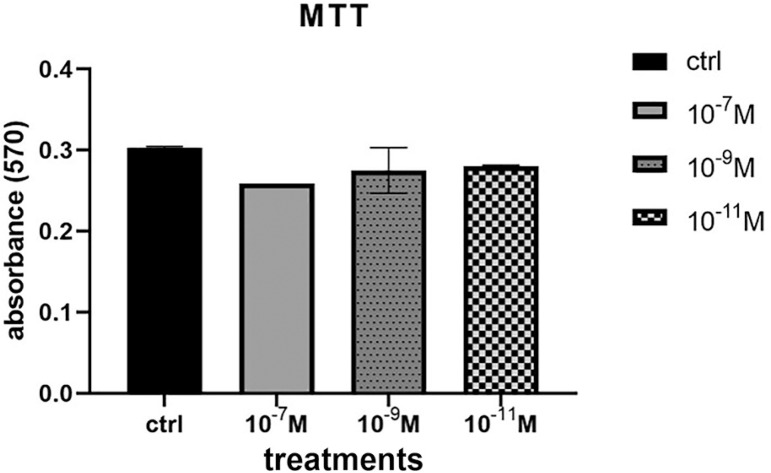
Viability of FF cells in control and groups treated with melatonin 10^-7^M, 10^-9^M and 10^-11^M for 1 week. Cell viability did not change significantly in the four groups.

Trypan blue staining confirmed that nearly all cells transferred from the hospital to the laboratory survived. Microscopy images showed that unstained cells were viable and that the dye did not pass through cell membranes. The various concentrations of melatonin were assessed for cytotoxic or proliferative effects on human FFMSC. MTT assay analysis showed that melatonin did not change cell viability compared to the control group (Figure 2).

### Flow cytometry

**Figure 3:**
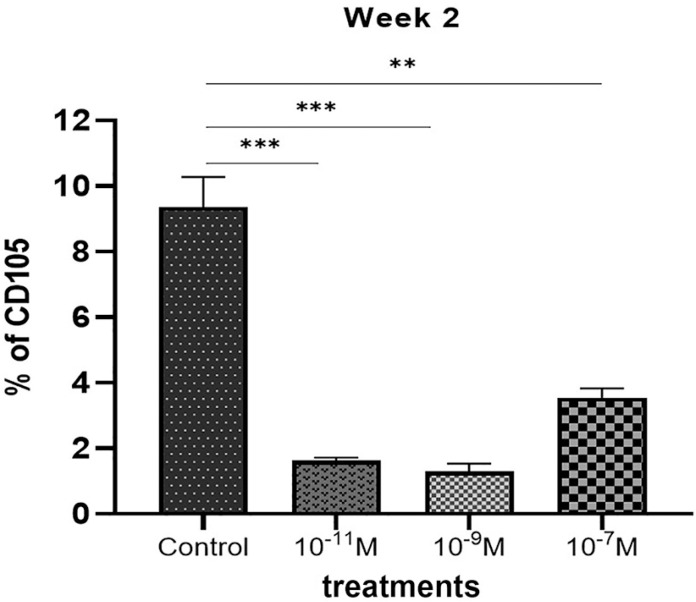
Flow cytometry analysis of CD105 antigen expression in the control group compared with melatonin treated groups after 2 weeks. High significant expression. observed in control group confirming the most stemness characteristic of the cells without melatonin treatment. **<0.01; ***<0.001.

CD105 antigen expression after 2 weeks of culture was evaluated in the four groups. Significant positive expression was observed in the control group in comparison with the melatonin-treated groups. The most significant CD105 expression levels among treated groups were observed in the 10^_7^M melatonin-treated group (Figure 3).

### Immunocytochemistry staining

**Figure 4:**
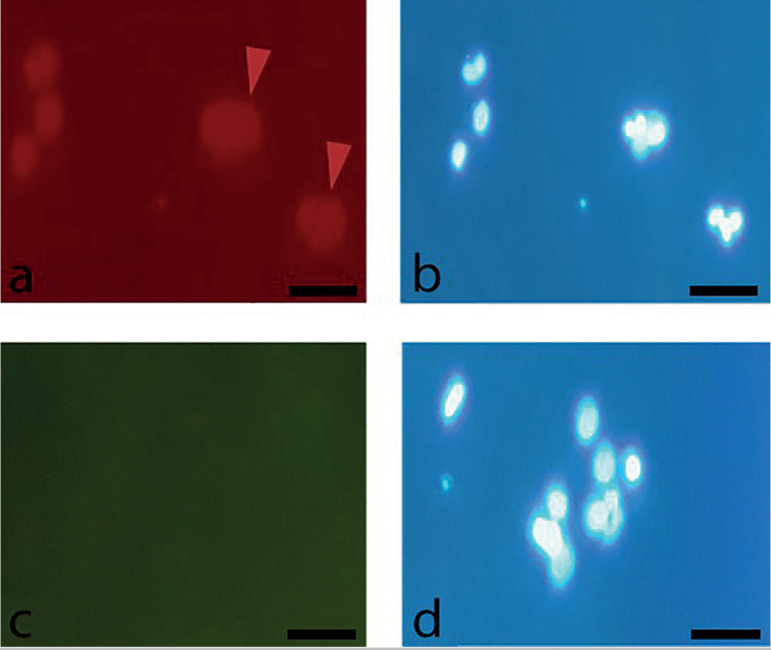
Immunostaining of human FF cells for specific cell surface markers after 48h. (a) Red arrows show cells low positive expression of CD105, (c) negative expression of CD34. Cell nuclei were counterstained by DAPI (b, d); 20X.

After 48h of culture, adherent cells showed low positive staining for CD105 and negative staining for CD34 as a hematopoietic stem cell marker (Figure 4).

### Adipogenic and osteogenic differentiation potential

**Figure 5:**
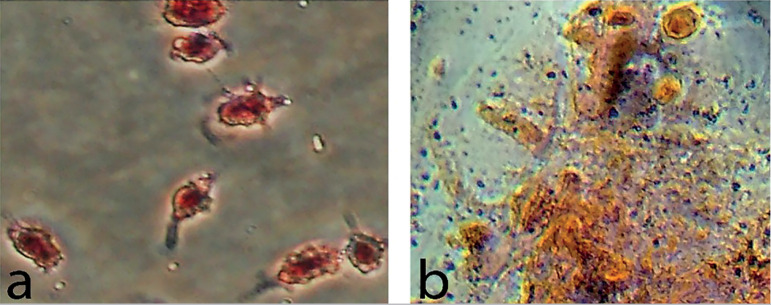
Adipogenic and osteogenic differentiation of human follicular ﬂuid cells. (a) Adipogenic differentiated cells of human FF showed by Oil Red O staining after 2 weeks. (b) Cells differentiated with osteogenic medium showed brownish red after staining with Alizarin Red; 20X.

Evaluation of the differentiation potential of the follicular aspirate cell population into adipogenic and osteogenic lineages was performed. Cells showing adipogenic differentiation stained positively with Oil Red O dye and cells showing osteogenic differentiation were recognized by brownish red Alizarin Red staining (Figure 5).

### Clonogenic Capacity

**Figure 6:**
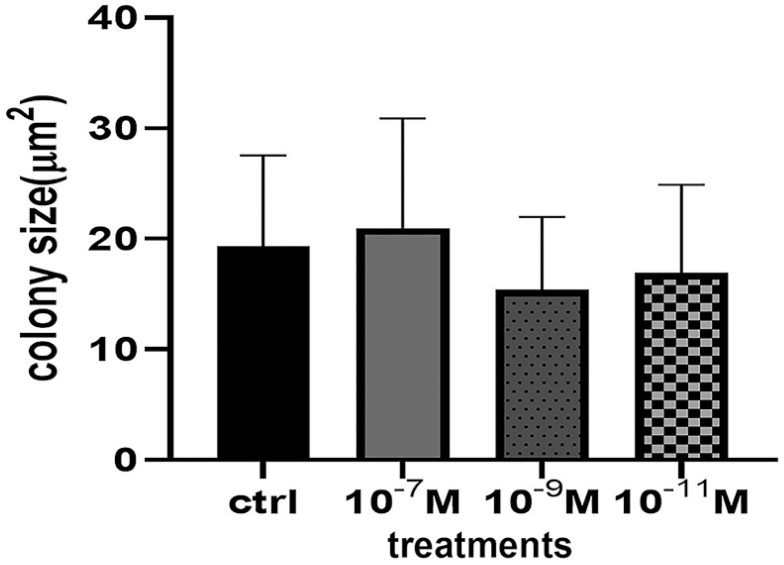
Colony forming unit fibroblast assay analysis of human FF cells after 2 weeks. Colony forming did not show significant differences between control and treated groups.

One of the characteristics of MSCs is that they generate colonies from a single living cell. After 14 days of culture in minimum conditions, observations showed that CFU-F sizes did not change significantly with melatonin treatment (Figure 6).

### qRT-PCR

**Figure 7:**
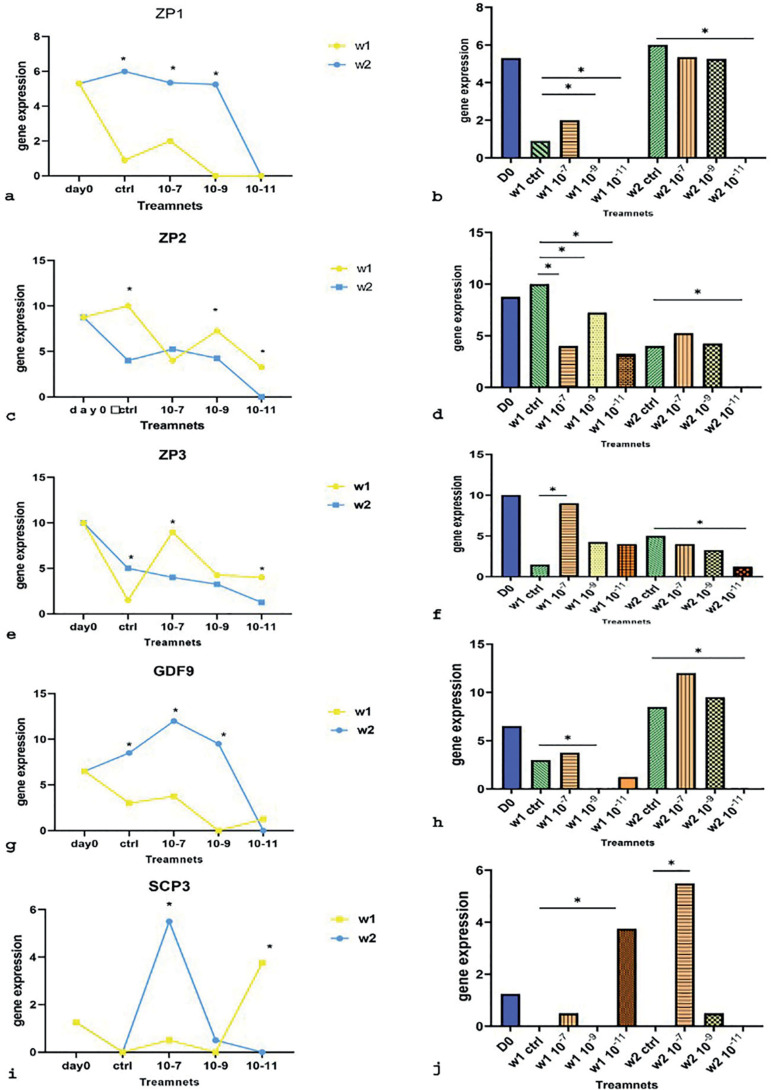
qRT-PCR analysis of ZP1(a, b), ZP2(c, d), ZP3(e, f), GDF9(g, h) and SCP3(I, j). mRNA expression. of human FF-derived OLCs on day 0, week 1(w1) and week 2(w2) in melatonin treatment groups (10^-7^, 10^-9^,10-^11^M) compared with control groups; gene expression at a specific concentration in week 1 compared to week 2 (a, c, e, g, i); gene expression of melatonin treatment groups compared with control groups each week (b, d, f, h, j); *: *p*<0.05.

qRT-PCR analysis was performed to compare ZP1, ZP2, ZP3, GDF9 (oocyte specific marker) and SCP3 (meiosis specific marker) gene expression levels at different doses of melatonin during a period of 2 weeks. ZP genes showed different expression patterns. Except for the 10^-11^M melatonin-treated group, ZP1 expression increased noticeably on the second week. The highest expression of ZP1 in the first week was observed in the group treated with melatonin 10^-7^M, which showed a significant increase compared to other treatment groups, although the difference was not significant compared to the control group. The lowest expression on this week was observed in the groups treated with melatonin 10^-9^M and 10^-11^M. In the second week, the highest non-significant gene expression levels were observed in the group treated with melatonin 10^-7^ M compared to other treatment groups. The lowest expression levels were observed in the group treated with melatonin 10^-11^M (Figure 7 a, b). ZP2 decreased mainly in week 2 compared to week 1. In the first week, the highest expression of ZP2 was observed in the group treated with melatonin 10^-9^ M compared to the control group. The lowest expression levels were observed in the group treated with melatonin 10^-11^ M compared to the control group. In the second week, the highest gene expression levels were observed in the group treated with melatonin 10^-7^ M compared to the control group, although this increase was not statistically significant. The lowest significant expression levels this week were observed in the group treated with melatonin 10^-11^M. (Figure 7 c, d). ZP3 expression levels dramatically increased in the group treated with melatonin 10^-7^M in week 1. In week 2, expression levels decreased compared to week 1 except for the control group. In the first week, the highest expression levels of ZP3 gene were seen in the group treated with melatonin in 10^-7^M. The lowest gene expression levels in week 1 were observed in the control group. In the second week, the highest and lowest gene expression levels were observed in the control group and in the group treated with melatonin 10^-11^ M, respectively. In this week, it was shown that ZP3 expression decreased as melatonin concentrations decreased. (Figure 7 e, f). The expression of GDF9 in week 2 was recognizable, except for the group treated with melatonin 10^-11^M. In the first week, the highest expression levels of GDF9 were observed in the group treated with melatonin 10^-7^ M and the lowest in the group treated with melatonin 10^-9^ M compared to the control group. In week 2, the highest GDF9 expression levels were seen in the group treated with melatonin 10^-7^ M. The lowest gene expression levels were seen in the group treated with melatonin 10^-11^M (Figure 7 g, h). SCP3 showed a significant increase in week 2, with the group treated with melatonin 10^-7^M showing the highest expression levels compared to other groups. In week 1, a significant increase was observed in the group treated with melatonin 10^-11^ M compared to the control group. (Figure 7 i, j).

### Hormone levels

**Figure 8:**
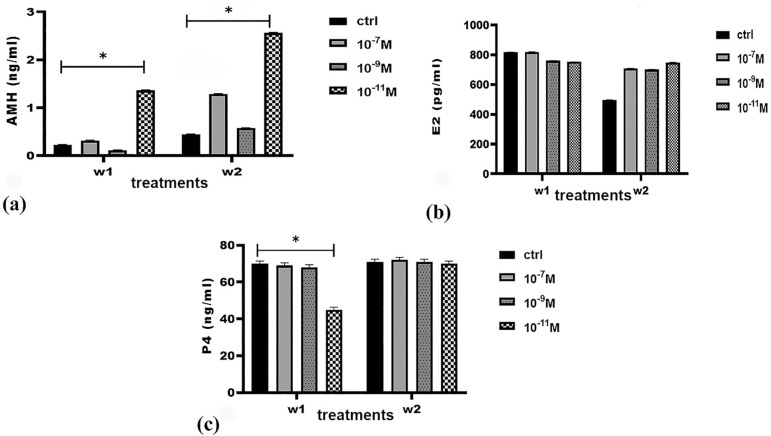
AMH (a), Estradiol (b), and Progesterone(c) production during FF cell culture in vitro. Hormone levels compared between control and melatonin treated groups (10^-7^, 10^-9^, 10^-11^) in 2 weeks; *: *p*<0.05.

AMH levels increased significantly in the group treated with melatonin 10^-11^M in both weeks. Estradiol levels did not change significantly in the groups treated with melatonin. Progesterone levels increased only in the group treated with melatonin 10^-11^ M in week 2 compared to week 1; significant changes were not observed in the other groups (Figure 8).

## DISCUSSION

Human adult stem cells are being evaluated for various stem cell-based therapies. Among these cells, MSCs have been considered for different applications because of their capacity to self-renew and in-vitro manipulation possibilities (Rubio *et al*., 2005; Bobis *et al*., 2006). The use of MSCs from human follicular fluid aspirate based on the International Society for Cellular Therapy (ISCT) criteria has been validated (Samsonraj *et al*., 2017). Fibroblast-like cells derived from FF changed their spherical shape and became adherent to plastic surface. The ability of MSCs to differentiate into other lineages is a marker of the multipotential nature of these cells, as demonstrated by their differentiation into adipocytes and osteocytes. To complete the characterization of the FF stem cell population, the expression of CD105 and lack of CD34 expression were also determined. Immunofluorescence staining showed low positive expression for CD105 as described by Tsai *et al*. (2004). It is known that indirect staining signal amplification provided by secondary antibodies can be more sensitivity than the direct method (Becheva *et al*., 2018). In a study on human MSCs, researchers used indirect immunostaining and reported a high positive expression levels for CD105, as expected (Haasters *et al*., 2009).

Clonogenic capacity assessment showed that cells might be cloned from follicular ﬂuid with features of MSCs. Although larger colonies were observed in the group treated with melatonin 10^-7^M, the difference in size was not significant. Previous reports showed that CFU-F did not change in groups treated with melatonin compared to controls. Older age and oxidative stress did not change colony sizes (Stolzing & Scutt, 2006; Pochampally, 2008). Contrary to our findings, some authors described increasing oxidative stress levels in smaller colonies. Various reasons may lead to this difference, including the stem cell isolation protocol, the culture medium used, and the sampling method (Stenderup *et al*., 2003; Stolzing & Scutt, 2006).

In this experiment, the MTT assay was used as a comprehensive method for measuring cell viability and melatonin toxicity. Phonchai *et al*. (2019) found that melatonin did not affect cell survival by examining different concentrations of melatonin on mesenchymal stem cells. Conversely, Rafat *et al*. (2019) reported cell viability increases in groups treated with melatonin compared to controls. The authors described a beneficial role for melatonin in the viability of both adipose-derived and bone marrow-derived mesenchymal stem cells. These differences may be due to different cell origins and different melatonin concentrations used in each study.

Melatonin as a protective hormone from oxidative damage plays an important role in regulating the physiological function of stem cells (Wang *et al*., 2019). It is generally accepted that high melatonin levels in follicular fluid suggest a potential beneficial property of melatonin by direct effects on reproductive organs (Yang *et al*., 2020). It has been described that the effects of melatonin as a concentration-dependent antioxidant might vary depending on the stage of oocyte development (Wu *et al*., 2019). In the present study, melatonin did not affect cell viability or the proliferation of human FF stem cells, as reported by Phonchai *et al*. (2019). However, Rafat *et al*. (2019) showed increasing cell viability with melatonin treatment. This discrepancy may be ascribed to differences in the origin of MSCs or melatonin concentrations in each study. Researchers have tried to find a dose of melatonin that might be more effective for OLCs extracted from human follicular during in vitro maturation. Treatment with melatonin 10^-7^ M has been associated with the attainment of more OLCs with the best morphology. Tian *et al*. (2014) found that the best melatonin concentrations for in vitro bovine oocyte maturation ranged from 10^-9^ M to 10^-7^ M. Another study by Adriaens *et al*. (2006) indicated that effective melatonin concentrations for mouse follicle maturation should be of no less than 10^-5^M.

This study examined the role of different concentrations of melatonin (10^-7^M, 10^-9^ M, and 10^-11^M) on the expression pattern of ZP1-3, GDF9, and SCP3 genes involved in oocyte maturation in weeks 1 and 2. Our results revealed positive correlations between SCP3 and GDF9 expression and melatonin. The expression of these two genes increased in the groups treated with melatonin 10^-7^M. As a result, more OLCs might develop. It has been suggested that increasing the expression of these two genes along with oocyte growing requires higher melatonin concentrations. The expression of ZP1 and ZP3 genes showed a significant increase in the group treated with melatonin 10^-7^M in the first week. Higher ZP2 expression levels were observed in the first week, except for the group treated with melatonin 10^-7^M. The low gene expression levels seen at low melatonin concentrations (10^-11^M), notably in the second week, likely indicates the expression of genes and consequently the maturation of OLCs depending on the presence of melatonin as a supplement in the culture medium. One might say that low concentrations of melatonin have an inhibitory effect on gene expression compared to controls (Zhao *et al*., 2018). Overall, the positive effect of high melatonin concentrations reported in this study cannot be ignored. However, to complete these findings, more research is needed with individuals with similar characteristics and more appropriate concentrations of melatonin.

The extent of differentiation of mesenchymal stem cells may be investigated through oocyte gene expression in these cells. Evidence suggests that during human folliculogenesis, ZP genes are only expressed in oocytes (Gook *et al*., 2008). This has been well demonstrated in the present study in all groups treated with melatonin and controls, as mesenchymal stem cells became OLCs capable of expressing ZP genes. Canosa *et al*. (2017) also reported that ZP expression in human oocytes depends on different factors such as maturity. By examining oocytes in metaphase I, metaphase II, and germinal vesicle stages, the authors found that expression of ZP1 and ZP2 in mature oocytes was significantly lower than in immature oocytes; changes in ZP3 expression were not significant. Cui *et al*. (2007) reported that the expression of ZP1, ZP2, and ZP3 genes in immature oocytes was higher than in mature oocytes. In another study, after culturing ovarian epithelial stem cells for 20 days, the authors obtained OLCs in which the expression of various genes was analyzed, including ZP2. ZP2 expression in OLCs was confirmed using RT-PCR. Other studies described the lack of expression of this gene in adult oocytes (Virant-Klun *et al*., 2013). Dalman *et al*. (2019) showed that gene expression changes throughout oocyte development, as exemplified by ZP1-3 on days 0, 18, and 25. Our study also showed a significant increase in ZP1 expression on day 25. Although the expression for these genes was incremental, the changes were not statistically significant. In the present study, ZP1 expression was higher in the second week than in the first week, which might indicate the immaturity of the OLCs. ZP2 expression was decreased in all groups, except for the one treated with melatonin 10^-7^M in the second week. Differently from controls, ZP3 expression was decreased in the treatment groups. These results possibly indicate the gradual evolution of OLCs. In agreement with previous studies, cells in the second week of the experiment had both mature and immature properties.

Another gene studied is GDF9, which biological and physiological activities have been studied alongside an essential role in female fertility. Abnormal expression of this gene is probably linked to infertility problems (Belli & Shimasaki, 2018). Some authors have demonstrated the role of GDF9 as a promoter of primary follicle growth involving fertility (Otsuka *et al*., 2011). Some authors studied this gene in humans and described expression in the early follicle stage. However, other authors consider its expression from the primordial stage onwards (Sun *et al*., 2010). A study described high expression of this gene on day 0 followed by decreased expression later on, after stem cells taken from human follicles were cultured and examined for GDF9 expression on days 0, 18, and 25 (Dalman *et al*., 2019). Additionally, according to a study on follicles taken from goats, the expression of GDF9 increased during follicular growth and the highest levels were observed in the secondary follicle. Having said that, its rate was reported to be similar in primordial and primary follicles (Almeida *et al*., 2011). In the present study, the expression of GDF9 in the second week, except for the group treated with the lowest concentration of melatonin (10^-11^ M), was higher, which indicates the development of OLCs.

Another gene studied is SCP3, a marker for meiotic division which absence promotes aneuploidy, trisomy, or monosomy linked to reproductive and fertility problems in general (Bolor *et al*., 2009). Dalman *et al*. (2019) examined the expression of SCP3 in the differentiation of human theca stem cells into OLCs. The authors described no expression of this gene on day 0; expression was higher on day 25 than on day 18, although not statistically. In another study, researchers isolated OLCs after 20 days of cell culture and examined the expression of SCP3. The lack of SCP3 expression in the cells was observed, which probably indicated the immaturity of these cells (Virant-Klun *et al*., 2008). Researchers also showed, by dividing OLCs into groups featuring large or small cells that SCP3 expressed only in large cells (Silvestris *et al*., 2019). The results of the present study also showed higher levels of expression of this gene in the second week in the group treated with melatonin 10^-7^ M, which was a significant increase compared to the first week and controls. This finding suggests that cells treated with melatonin 10^-7^M were more differentiated after 2 weeks than the cells in other study groups.

Since culture medium compounds may indicate the viability and growth potential of oocytes, the levels of some hormones were also measured. According to this study, cells in DMEM-HG culture medium can produce estradiol, progesterone, and AMH in the presence or absence of melatonin. It also showed that estradiol levels remained almost constant for two weeks in the presence of melatonin. Progesterone levels did not change significantly among the groups during this time, except for the decrease seen in the group treated with 10^_11^M melatonin in week1. AMH levels increased significantly in the group treated with melatonin 10^-11^ M in both weeks. Another study described low, constant levels of progesterone in mice gametogenesis culture medium until day 25, from which time they increased; estradiol levels were also low and constant until day 27, only to significantly increase from then on (Morohaku *et al*., 2016). Another study reported that estradiol levels in follicular fluid were almost constant in small follicles. But in larger follicles, levels increased with follicle diameter. Mean progesterone levels in follicles, especially in the larger ones, were positively correlated with follicle diameter (Andersen *et al*., 2010). According to these studies, one might conclude that cells in culture medium containing melatonin were not mature enough to produce more of these two hormones and might need more time to do so. Studies have also shown that AMH is an important hormone in ovarian follicle development. According to some authors, AMH is present only in primary, secondary, and antral follicles. Other authors have reported the presence of AMH in primordial follicles (Weenen *et al*., 2004; Stubbs *et al*., 2005; Rocha *et al*., 2016). Previous studies suggested that the presence of AMH in the culture medium of human preantral follicles produced an inhibitory role in follicular activation (Carlsson *et al*., 2006). Andersen *et al*. (2010) described an inverse relationship between follicle diameter and intrafollicular concentration of AMH. The authors reported a sharp decrease in AMH levels at follicular diameters greater than 9 mm, thus corroborating the idea that AMH is an important mediator of folliculogenesis. Oocyte secreted factors are likely to play a significant role in AMH levels. La Marca & Sunkara (2014) described AMH as a good criterion for predicting the quality of oocytes and embryos for IVF treatments. Researchers examined follicular fluid AMH levels and oocyte quality. They reported AMH levels were negatively correlated with IVF outcomes and estradiol levels (Mehta *et al*., 2013). AMH levels in preantral follicles are almost three times greater than the levels seen in antral follicles. AMH levels have been positively associated with progesterone levels in contrast to estradiol levels (Andersen & Byskov, 2006). In this study, an increase was observed in AMH levels in the group treated with melatonin 10^-11^ M. This might indicate the inhibitory role of AMH production in low concentrations of melatonin in OLC development.

Our findings indicate that follicular fluid is a source of MSCs that can differentiate into OLCs under culture medium conditions. We found out that melatonin at a proper concentration might help the maturation of oocyte-like cells. This study also indicated oocyte-like cells undergo differentiation and have both oocyte and stem cell properties.
